# Regular cannabis use is associated with altered activation of central executive and default mode networks even after prolonged abstinence in adolescent users: Results from a complementary meta-analysis

**DOI:** 10.1016/j.neubiorev.2018.10.026

**Published:** 2019-01

**Authors:** Grace Blest-Hopley, Vincent Giampietro, Sagnik Bhattacharyya

**Affiliations:** a Department of Psychosis Studies, Institute of Psychiatry, Psychology & Neuroscience, King’s College London, UK; b Department of Neuroimaging, Centre for Neuroimaging Sciences, PO Box 089, Institute of Psychiatry, Psychology & Neuroscience, King’s College London, UK; c South London and Maudsley NHS Foundation Trust, Denmark Hill, Camberwell, London, UK

**Keywords:** Cannabis, THC, Functional magnetic resonance imaging, Meta-analysis, Abstinence

## Abstract

Whether the effects of cannabis use on brain function persist or recover following abstinence remains unclear. Therefore, using meta-analytic techniques, we examined whether functional alterations measured using fMRI persist in cannabis users abstinent for over 25 days (or 600 h) as evidence suggests that the effects on cognitive performance no longer persist beyond this period. Systematic literature search identified 20 studies, of which, 12 examined current cannabis users (CCU) (361 CCU versus 394 non-cannabis using controls (NU)) and 3 examined abstinent cannabis users (ACU) in 5 separate comparisons (98 ACU versus 106 NU). Studies in ACU were carried out in adolescents and suggest significantly greater activation in components of the central executive and default mode networks in adolescent ACU compared to NU. While this evidence is to be interpreted with caution because studies were carried out in overlapping samples, they indicate a pressing need for independent confirmation whether certain neurofunctional alterations in adolescent cannabis users may persist even after cannabis and its metabolites are likely to have left their bodies.

## Highlights

• Adolescent abstinent cannabis users showed significantly greater activation in the dorsolateral and ventrolateral prefrontal and posterior parietal cortices compared to controls.
• Adolescent users showed increased activation in regions involved in executive functioning, attentional control and the default mode network compared to non-using controls.
• No significant group differences in brain activation observed between abstinent and current adolescent cannabis users.

## 1 Introduction

Cannabis use has been associated with changes in cognitive task performance (Curran et al., 2002; Jacobus et al., 2009; Schoeler et al., 2016a; Schreiner and Dunn, 2012a; Scott et al., 2018; Solowij and Pesa, 2010) and altered brain function (Batalla et al., 2013; Tapert et al., 2007) involving various cognitive domains. Impaired task performance (Bhattacharyya et al., 2015a; Curran et al., 2002; D’Souza et al., 2004; Hindocha et al., 2015, 2017; Lawn et al., 2016; Ramaekers et al., 2006) and brain functional alterations (Batalla et al., 2014; Bhattacharyya et al., 2017, 2015b) involving different cognitive domains have also been observed during acute intoxication. Recent meta-analyses employing different analytic approaches have shown that persistent long-term use of cannabis is associated with functional alterations in key brain regions across different cognitive tasks (Blest-Hopley et al., 2018; Yanes et al., 2018; Yücel et al., 2008).

One of the key issues that can potentially confound the interpretation of current evidence is whether the effects of cannabis use on cognition and underlying brain function abstinence persist or recover following a period of abstinence. Following abstinence, cognitive performance has been found to improve in cannabis users (CU) (Hanson et al., 2010) to the level of controls after longer periods of abstinence (Schulte et al., 2014), with cognitive deficits possibly only detectable within the first 25 days of abstinence. Meta-analysis of cognitive task performance in continuing CU has shown significant impairment over a wide range of tasks, while abstinent users showed no significant difference to controls in any specific or global cognitive domain (Schreiner and Dunn, 2012b). In contrast, structural changes in CU have been observed (Batalla et al., 2013), in particular decreased volume in the hippocampus (Chye et al., 2018; Cousijn et al., 2012; Matochik et al., 2005), that persisted after a prolonged abstinence in some (Ashtari et al., 2011) but not all studies (Koenders et al., 2017).

However, whether the effects of recreational cannabis use on brain function persist not only beyond the acute intoxication stage typically lasting 2–3 h (Grotenhermen, 2003)(when used by the inhalation route), but even after the key metabolites of delta-9-tetrahydrocannabinol (THC) with psychotropic effects have been excreted from the body, is less well known. THC and its metabolites(11-hydroxy-Δ9-tertrahydrocannabinol and 11-nor-9-carboxy-Δ9-tertrahrdrocannabinol (Sharma et al., 2012) are of particular interest, as it is the main psychotropic ingredient in cannabis known to be associated with harmful effects on various cognitive domains(Pertwee, 2008). It is worth noting that the half-life of THC in frequent users is 5–13 days (Smith-Kielland et al., 1999) and THC is detectable in urine for up-to 2–4 weeks(Lowe et al., 2009). Interestingly, the upper limit for the period of detection of metabolites in urine is consistent with the period over which cognitive deficits are detectable following abstinence (Schreiner and Dunn, 2012b).

We have recently examined the residual effects of recreational cannabis use on brain function in adult and adolescent cannabis users by meta-analytically combining the data from 20 published studies employing functional MRI techniques (Blest-Hopley et al., 2018). While some of these studies investigated cannabis-using participants after a period of abstinence, several others allowed cannabis use up until, as short a period as, 3 h prior to scanning. Therefore, interpretation of the results of these studies may be confounded by residual acute effects of THC and its metabolites that may still be left in cannabis-using participants as well as effects of withdrawal from cannabis. On the other hand, brain functional alteration following a sustained period of abstinence has also been investigated, though the results of these studies are less consistent, with users showing both increased (Chang et al., 2006; De Bellis et al., 2013; Jacobsen et al., 2004; Schweinsburg et al., 2008; Tapert et al., 2007) and decreased (Chang et al., 2006; Schweinsburg et al., 2008) activation compared to controls. Studies comparing cannabis users with different periods of abstinence have found greater activation in the prefrontal cortex and insula in recently abstinent users compared to users with longer (at least 27 days) periods of abstinence, who in turn had greater activation in the precentral gyrus (Schweinsburg et al., 2010). Another study that investigated cannabis users at multiple time-points following abstinence, reported that 28 days of abstinence resulted in reduced activation difference to controls in some regions, but some differences in brain activation persisted (Pillay et al., 2008). Therefore, understanding differences in brain activation between currently using (or non-abstinent) and abstinent cannabis users (ACU), is of particular interest. However, to our knowledge existing evidence in this regard has not been systematically reviewed and summarized using meta-analytic approaches. Hence, we have carried out a meta-analysis complementary to that previously reported by us (Blest-Hopley et al., 2018) to investigate whether altered brain function associated with regular cannabis use persists even after a sustained period of abstinence from cannabis. Consistent with our approach previously (Blest-Hopley et al., 2018), we included fMRI studies that employed a wide range of cognitive activation paradigms engaging various cognitive processes rather than focusing only on task-specific approaches as in other work (Yanes et al., 2018). Our strategy was driven by two key considerations. Firstly, only a limited number of available studies have specifically employed comparable activation paradigms limiting our ability to meaningfully investigate the question of interest here. More importantly, as we have argued before (Blest-Hopley et al., 2018), the effects of cannabis use are unlikely to be limited to only those brain regions that sub-serve cognitive processes examined in studies conducted hitherto. Rather they are more likely to be widely distributed, consistent with ubiquitous distribution of cannabinoid receptors in the brain (Iversen, 2003). Therefore, we included fMRI studies employing a range of cognitive activation paradigms to investigate using a meta-analytic approach whether brain functional alterations associated with cannabis use persist even after periods of abstinence sufficiently long such that cannabis metabolites are no longer detectable in urine.

## 2 Methods

### 2.1 Study identification

A systematic search was competed on the 13/12/2017 following the Cochrane Handbook (JPT, 2011) and the MOOSE (Stroup et al., 2000) guidelines, using the database PubMed. Two categories of search terms were used: 1) cannabis, marijuana, marihuana, THC, tetrahydrocannabinol and 2) imaging, fMRI, functional activation, BOLD. Following screening through abstract to meet inclusion criteria.

Inclusion criteria were:

• A published peer-reviewed manuscript reported in English
• A data-based publication
• Comparison of cannabis users to a non-cannabis using control group (NU) using fMRI
• Reported whole-brain imaging analysis results and not just region-of-interest analysis results
• Used a cognitive or emotional activation task with no cannabis-related stimuli

**Fig. 1:**
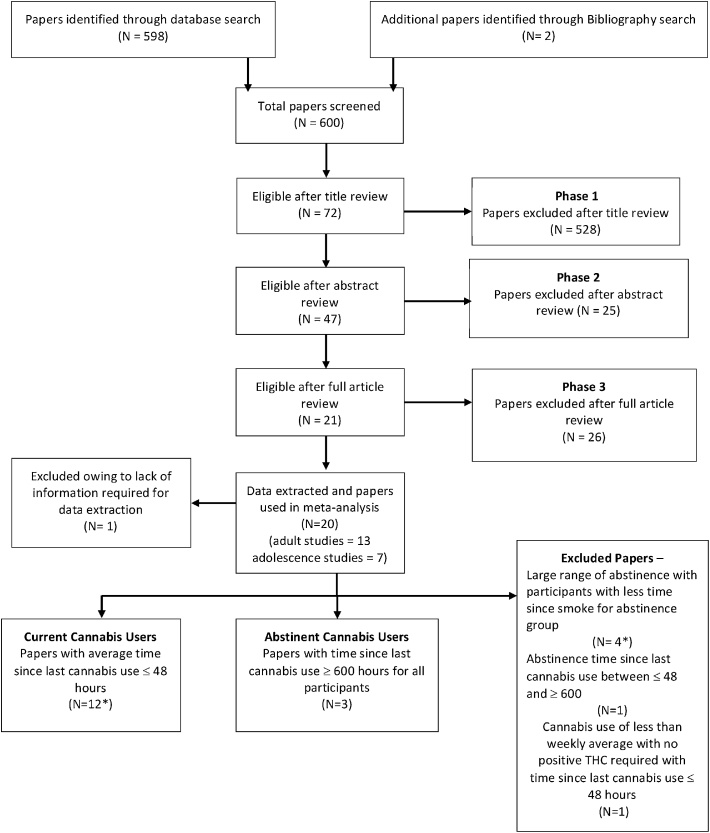
Flow-chart showing the identification, classification and inclusion of papers selected for meta-analysis. *One paper included two studies, one of which was eligible for inclusion, one of which was excluded.

Manuscripts were separated into three categories, based on the type of cannabis-using groups reported. Those that reported at least one comparison with current cannabis users (CCU) or with abstinent cannabis users (ACU) were included while those that did not report either of the previous comparisons were excluded (flow-chart in Fig. 1; number of manuscripts indicated by ‘N’). CCU were identified as those in whom the time interval between the last cannabis smoke and scanning was a maximum of 48 h (so as to avoid peak withdrawal symptoms (Budney et al., 2004)) with inclusion criteria requiring at least an average weekly use of cannabis or a positive test for THC or its metabolites at urine drug screening. ACU were required to have a monitored period of abstinence from cannabis use for at least 600 h/25 days based on previous evidence of upper limit of period of detection of THC metabolites in urine (Schreiner and Dunn, 2012b) and to have provided a negative urine test for THC.

### 2.2 Data extraction

Data extraction and data analysis using seed-based *d* mapping (SDM)(Sdmproject.com, 2017) was conducted as outlined previously (Blest-Hopley et al., 2018) and are described here in brief. Significant peak coordinates were extracted from included studies along with their t-statistic. In papers reporting z- values or p –values, t- statistics were computed using a converter provided with SDM (www.sdmproject.com/utilities /?show = statistics). In case of studies where no inferential statistical values were reported, a ‘p’ or ‘n’ was used to indicate a positive or negative peak, respectively. As per protocol for SDM meta-analysis (Radua et al., 2012) a study-specific text file was created for each study included in the meta-analysis, including the coordinates reported, t-value, and the number of participants for each group. In this file, the sign of the t-statistic was positive or negative, depending on the comparisons of interest. For example, for comparisons between CCU and healthy controls, greater activation in CCU compared to healthy controls was indicated as a positive t-statistic and vice versa. Similarly, for comparison of ACU with healthy controls, greater activation in ACU compared to healthy controls was indicated as a positive t-statistic and vice versa. Following this we tested whole-brain differences in activation between CCU and ACU by calculating the difference between both groups in each voxel and determining its statistical significance using a randomization test (Radua et al., 2010).

Information regarding the brain template used to report cluster coordinates (for example, Montreal Neurological Institute (MNI) or Talairach) was included in each study-specific file text file created as above. Any study that reported no significantly different activation peaks was also included. Each contrast completed between CU (CCU or ACU) and controls was extracted and treated as a separate studies, for example if a study reported the results of separate encoding and recall conditions in a memory task, these were treated as two separate studies, following established protocol (Radua et al., 2012). Each study was then assigned as a study with current (CCU) or abstinent (ACU) users.

### 2.3 Data analysis

Meta-analysis was carried out using seed based-d-mapping (Sdmproject.com, 2017), using the methods previously outlined (Radua et al., 2014). For voxels that contained a peak coordinate, the unbiased effect-size and variance were computed using standard formulae (Hedges and Olkin, 1985), while for all other voxels, the effect-size was estimated based on their distance to nearby peaks, using a 20 mm full-width-at-half-maximum non-normalized Gaussian kernel (Radua and Mataix-Cols, 2009). For voxels that were assigned a value from more than one peak coordinate, an average value was estimated by weighting by the square of the distance to each close peak. To reduce bias from a publication reporting numerous closely located peaks, a study maximum value was employed. Both the positive and negative activations were assigned to the same map. For coordinates with no t-value, a threshold-based imputation of effect-size was carried out by estimating the mean effect-size of peaks from studies that did report t-values, separately for each significance threshold. Individual effect-size maps were created for each study and a random effects model meta-analytically combined the data from each study, by weighting each study with the inverse of the sum of its variance plus the between-study variance as obtained by the DerSimonian-Laird estimator (DerSimonian and Laird, 1986). This has been shown to be statistically comparable to the restricted maximum likelihood (Viechtbauer, 2005). All maps were then included in to a meta-analysis seed-based d map, where a null distribution of the meta-analytic values was created to test which voxels had studies reporting activation difference around them by chance, using monte-carlo randomizations. Due to previous work yielding highly stable results with 20 randomizations (Radua et al., 2012), we carried out 20 randomisations for each meta-analysis. The co-ordinates of cluster peaks were then reported using MNI coordinates.

Three initial meta-analyses were completed comparing CCU to NU; comparing ACU to NU and, comparing activation in CCU to ACU. Two further meta-analyses were completed, as the ACU group contained only adolescent participants, comparing adolescent CCU compared to adolescent NU and comparing adolescent CCU to the adolescent ACU. Results have been thresholded to ensure voxel threshold = p < 0.005, peak height threshold: peak SDM-Z < 1, and a cluster-size threshold of clusters ≥ 10 voxels. The co-ordinates of cluster peaks were reported using MNI coordinates.

### 2.4 Assessment of study heterogeneity and publication bias

Heterogeneity Q statistic was assessed in terms of a chi-squared distribution after conversion to standard z values and reported. Between–study heterogeneity was assessed by comparison of heterogeneity maps. Funnel plots were created for each cluster peak and Egger’s test performed in order to asses publication bias (Sedgwick and Marston, 2015).

### 2.5 Assessment of study quality

Quality assessment of each study was completed using criteria previously used for fMRI studies (Radua et al., 2015), which we have reported before (Blest-Hopley et al., 2018).The quality assessment reported here has been amended to also include assessment of the extent to which studies accounted for use of substances other than cannabis. No studies were excluded from analysis based on this quality assessment. Studies that matched groups on use of substances other than cannabis scored 2 points, while those that did not study matched groups but instead used statistical methods to control for group differences in use of substances other than cannabis scored 1 point. Finally, certain studies controlled for only some substances and were rated 0.5 and studies that neither matched participant groups based on use of substances other than cannabis, nor controlled for them analytically were rated 0.

## 3 Results

### 3.1 Included studies

Twenty studies were identified as meeting study inclusion criteria as detailed earlier. Of those, twelve manuscripts met inclusion criteria as reporting CCU, with 22 separate comparisons, comparing 361 CCU to 394 NU (Table 1). Three manuscripts were identified as having studied ACU with 5 separate comparisons, comparing 98 ACU to 106 NU (Table 1). Five manuscripts and one comparison from another manuscript were excluded as they had large ranges of abstinence within their cannabis using group (Chang et al., 2006; Heitzeg et al., 2015; Jager et al., 2013; Nestor et al., 2010, 2008; van Hell et al., 2010) or had abstinent periods (between 48 and 600 h) that were not consistent with our pre-defined abstinence criteria for inclusion in the ‘abstinent users’ group. Number of studies included for each comparison are indicated by ‘k’ and number of participants for each comparison are indicated by ‘n’ henceforth in the text as well as in the relevant sections of tables or figures.

**Table 1:** Studies included in meta-analysis.

Current Cannabis User Studies	fMRI activation task	CU M/F	NU M/F	Age of CU (years)	Age of NU (years)	Quantity of cannabis used by CU	Time between scan and last smoke *	Age of onset of cannabis use for CU (years)	Average years of cannabis use by CU	Task condition	Results whole brain analysis	Task Performance results	Number of task comparisons	Tesla
Abdullaev et al., 2010	Attention Network Task	10/4	10/4	19.5 (0.8) (SD)	19.7 (1.4) (SD)	71-196 days per year	48	12-16	N/A	Executive task; Alerting task; Orienting task.	CU > NU R-LPFC, supplementary motor cortex, Lateral parietal cortex; No difference for alerting & orientation task.	Longer reaction time for CU. More errors made for executive task.	3	3T
	Use Generation Task	5/2	5/2	19.6 (0.9) (SD)	20 (0.2) (SD)	71-196 days per year	48	12-16	N/A	Generating nouns versus reading nouns; difficult words versus easy words.	CU > NU R-VPFC NU > CU Bi -ACG to L-PFC, L- TPC; CU > NU R - ACC, R FOC, L frontal pole & L precuneus.	N/A	2	3T
Smith et al., 2011	Go/NoGo Task	6/4	9/5	19-21	19-21	> 1 joints per week	3	N/A	4.55 years	Press all but X; Press X	No significant differences in both tasks after including covariates.	No Significant difference	2	1.5T
Chang et al., 2006	Visual- Attention Task	9/3	11/8	27.91 6 3.13 (SEM)	30.57 6 1.83 (SEM)	≥5 days per week	4	9–20	36–448 months	Visual attention	CU > NU small clusters of L precuneus, L-LG & L limbic uncus. NU > CU R-FC, Bi- dorsal parietal and R cerebella.	No Significant difference	1	4T
Cousijn et al., 2012	Iowa Gambling Task	21/11	26/15	21.4 (2.3) (SD)	22.2 (2.4) (SD)	> 10 days per month	38.4	N/A	2.5 (1.9) (SD)	Win > Loss; Loss > Win	CU > NU R-OFC, R insula, L-STG; No activation difference.	No Significant difference	2	3T
Gruber, Rogowska and Yurgelun-Todd, 2009	Facial effect task	14/1	14/1	25 (±8.8)	26 (±9.0)	4-7 days per week	12	14.9 (±2.50)	N/A	Viewing Angry; Viewing Happy	CU > NU R-IFG, R-precuneus, R-paracetral lobe, L-SFG, cerebellar, R-MiTG, NU > CU L-SPL, interhemispheric precuneus, L- CG ; CU > NU cerebella, NU > CU L -STG & sub.lobular space.	No Performance data	2	3T
Heitzeg et al., 2015	Emotional arousal word task	12/8	14/6	19.84 (1.45) (SD)	20.51 (1.26) (SD)	>100 time (average 618.12)	48	N/A	13.4 (2.7) (SD)	Negative words; Positive words.	NU > CU R- MiFG, R- DLSFG, R- MiTG, R-STG, R calcarine fissure, R- L, insula CU > NU R Dorsolateral SFG, NU > CU R-IPL.	No Significant difference	2	3T
King et al., 2011	Checker-board task	16/14	16/14	M = 21 F = 22.5	M = 23 F= 24.5	6-7 days per week	12	M = 14.5 F = 16 (years)	M = 78 F = 63 (months)	2HZ frequency; 4HZ frequency	CU > NU SFG, NU > CU LG & cuneus; L- postcentral gyrus, Bi- MiFG, R-SPG, R- frontal pole, NU > CU R- postcentral gyrus, R- precentral gyrus & L- LG.	None Taken	2	3T
Kanayama et al., 2004	Spacial working memory Task	10/2	6/4	37.9 (7.4) (SD)	27.8 (7.9) (SD)	5100-54000 life time use	21	N/A	>5000 lifetime use	Short- delay task minus perception task.	CU > NU R-SFG, L-MiFG, IFG, R-STG, Bi. ACG. R. precentral gyrus, Bi -caudate & R-putamen. NU > CU Bi -MiFC.	No Significant difference	1	1.5T
Wesley, Hanlon and Porrino, 2011	Iowa Gambling Task	9/7	6/10	26.4 (3.6) (SD)	26.6 (6.1) (SD)	Mean 29.4 days per month	12	16.3 (2.1) (SD)	9.6 (4.1) (SD)	Win; Lose	No difference in Win; NU > CU Bi. MFG, R ACC, R-Precuneus & R- SPL, L declive.	More loss events for CU	2	1.5T
Adolescent Current Cannabis Users	Task	CU M/F	NU M/F	Age of CU (years)	Age of NU (years)	Quantity of cannabis used by CU	Time between scan and last smoke *	Age of onset of cannabis use for CU (years)	Average years of cannabis use by CU	Trials	Results whole brain analysis	Task Performance results	Number of task comparisons	Tesla
Acheson et al., 2015	Win/Lose Gambling Task	11/3	11/3	17.3 (1.3) (SEM)	17.6 (1.0) (SEM)	>5 uses per week	12	N/A	N/A	Win ; Loss	CU > NU Bi- MiFG, caudate claustrum; CU > NU R- MiFG, R- PCC R- ACC, L-Insula, Bi. claustrum Bi- declive.	Not Reported	2	3T
Behan et al., 2014	Go/NoGo Task	16/1	17/1	16.5 (0.2) (SEM)	16.1 (0.4) (SEM)	42.9 mean joints per week	12	13 (0.2) (SEM)	N/A	Successful inhibition	NU > CU Bi. white matter adjacent to ACC.	CU significantly worse at inhibition task.	1	3T
Lopez-Larson et al., 2012	Finger Tapping	22/12	17/7	18.2 (0.7) (SD)	18.0 (1.9) (SD)	Mean use of 10.3 joints per week	24	15.3 (1.4) (SD)	N/A	Finger taping	NU > CU R- CG	Not Reported	1	3T
Abstinent Cannabis User Studies	Task	CU M/F	NU M/F	Age of CU (years)	Age of NU (years)	Quantity of cannabis used by CU	Time between scan and last smoke *	Age of onset of cannabis use for CU (years)	Average years of cannabis use by CU	Trials	Results whole brain analysis	Task Performance results	Number of task comparisons	Tesla
Schweins-burg et al., 2011	Verbal Encoding Task	27/9	29/9	18.1 (0.9) 18.0 (1.0) (SD)	17.6 (0.8) 18.1 (0.7) (SD)	480.7 (277.2 SD) life time use	600	14.5 (2.5) 14.9 (3.4) (SD)	N/A	Novel encoding	No significant difference.	No Significant Difference	1	3T
Schweins-burg et al., 2008	Spacial working memory Task	11/4	12/5	18.1 (0.7) (SD)	17.9 (1.0) (SD)	480.7 (277.2 SD) life time use	672	N/A	4.0 (1.6) (SD)	SWM> Viligance; Viligance> SWM.	CU > NU R- SPL NU > CU R- DLPFC; CU > NU R - Inferior cuneus.	No Significant Difference	2	1.5T
Tapert, et al, 2007	Go/NoGo Task	12/4	12/5	18.1 (0.7) (SD)	17.9 (1.0) (SD)	>60 times	672	14.0 (1.6) (SD)	N/A	Inhibition; Go	CU > NU Bi- SFG, Bi- MiFG, R-Insula, L- MPFC, Bi- PPC, R- LG, CU > NU R-IFG, R- insula, R-SFG, R-SPL, R-IPL, R medial precuneus.	No Significant Difference	2	1.5T

*Time between scan and last smoke reported here as the mean or median estimate (number of hours) reported in the manuscript, or based on the inclusion/exclusion criterion related to minimum period of abstinence reported in the manuscript.

CU = Cannabis users NU = Non-using controls, R = Right, L = Left, Bi = Bilateral, LPFC = Lateral Prefrontal Cortex, VPFC = Ventrolateral Prefrontal Cortex, DLPFC = Dorsolateral Prefrontal Cortex, MPFC = Medial Prefrontal Cortex, PFC = Prefrontal Cortex, OFC = Orbitofrontal Cortex, FC = Frontal Cortex, MFG = Medial Frontal Cortex, MiFG = Middle Frontal Gyrus, SFG = Superior Frontal Gyrus, DLSFG = Dorsolateral Superior Frontal Gyrus, FOC = Frontal Orbital Cortex, IFG = Inferior Frontal Gyrus, PPC = Posterior Parietal Cortex, IPL = Inferior Parietal Lobe, TPC = Temporo-Parietal Cortex, SPL = Superior Parietal Lobe, SPG = Superior Parietal Gyrus, MTG = Medial Temporal Gyrus, MiTG = Middle Temporal Gyrus, STG = Superior Temporal Gyrus, LG = Lingual Gyrus, CG = Cingulate Gyrus, ACC = Anterior Cingulate Cortex, ACG = Anterior Cingulate Gyrus.

Summary of quality assessment of studies included in the present meta-analyses are reported in Table 2. Although, no studies were excluded from analysis based on this quality assessment, as is evident from the summary, studies did not always control for the effect of substances other than cannabis that may have been used by study participants. However, it is worth noting that all of the adolescent ACU studies controlled for these effects statistically.

**Table 2:** Quality assessment.

Study	Sample size	Inclusion criteria	Exclusion criteria	Control for other substance use	Match for age/sex/ handedness/ education	Control for motion artefacts	Co-registration with anatomical image	Software and statistical test applied	Correction for multiple testing	Sum of the scores & category
Current Cannabis Users										
Abdullaev et al., 2010	1	1	1	2	1	2	2	2	2	14
Smith et al., 2011	0.5	1	1	1	2	2	2	1	2	13
Chang et al., 2006	1	2	2	0	2	2	2	2	2	15
Cousijn et al., 2012	2	2	2	1	2	2	2	2	2	17
Gruber, Rogowska and Yurgelun-Todd, 2009	1	2	1	1	2	2	2	2	0	13
Heitzeg et al., 2015	1	2	2	2	1	2	2	2	2	16
King et al., 2011	2	2	2	0.5	1	2	2	2	2	15.5
Kanayama et al., 2004	0.5	1	1	2	1	2	2	2	0	11.5
Wesley, Hanlon and Porrino, 2011	1	1	2	0.5	2	2	2	2	2	14.5
Adolescent Current Cannabis Users										
Acheson et al., 2015	1	2	1	0	1	2	2	2	2	13
Behan et al., 2014	1	1	1	0	2	2	2	2	2	13
Jager et al., 2013	2	2	1	1	1	0	2	1	2	12
Adolescent Abstinent Cannabis User Studies										
Schweinsburg et al., 2011	2	2	1	1	1	2	2	2	2	15
Schweinsburg et al., 2008	1	1	1	1	1	2	2	2	2	13
Tapert, Schweinsburg and Brown, 2008	1	1	1	1	2	2	2	2	2	14

**Rating criteria:**Sample size: n1 < 12,n2 < 12: 0 point; n1 < 12,n2 = 12-20: 0.5 point; n1 < 12,n2 > 20: 1 point; n1 = 12–20,n2 < 12: 0.5 point; n1 = 12–20,n2 = 12-20: 1 point; n1 = 12–20,n2 > 20: 1.5 point; n1 > 20,n2 < 12: 1 point; n1 > 20,n2 = 12-20: 1.5 point; n1 > 20,n2 > 20: 2 point. Inclusion criteria: 0 (not reported), 1 (partly reported), 2 (reported). Exclusion criteria 0 (not reported), 1 (only one reported), 2 (reported). Control for other substance use: Groups not matched for other substance use and not statistically controlled for 0 points; groups not matched for other substance use and only some substances statistically controlled for 0.5 points; groups not matched for other substance use, but statistically controlled for 1 point; groups matched for other substance use 2 points. Matched for age/sex/handedness/education: 0 (for no parameter), 1 (partly), 2 (for all parameters). Control for motion artefacts: 0 (not performed), 2 (performed). Co-registration with anatomical image: 0 (not performed), 2 (performed) Software and statistical test applied: 0 (not reported), 1 (partly reported), 2 (reported).

Correction for multiple testing: 0 (not corrected), 2 (corrected).

**Fig. 2:**
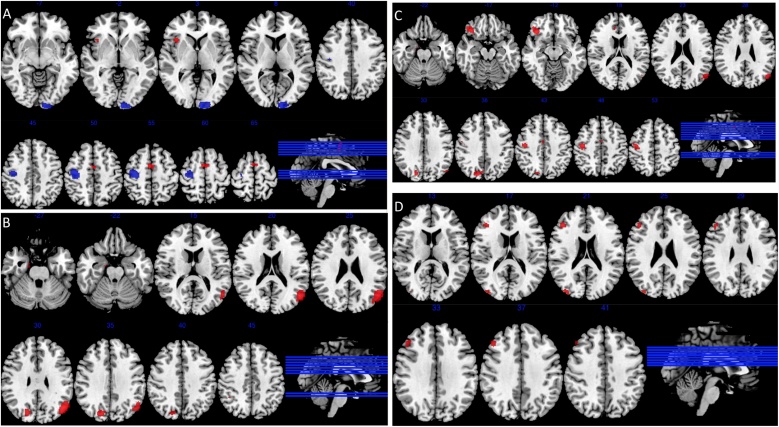
Maps of statistically significant differences in activation (Voxel threshold = p < 0.005, peak height threshold: peak SDM-Z < 1, clusters ≥ 10). Axial brain slice position shown on a sagittal view bottom right, with slices arranged from left to right in the different panels showing brain slices in ascending order from bottom to top. A - Activation of current CU compared to non-using control subjects, increased activation in CU shown in red, decreased activation in CU shown in blue (k = 22; CU n = 361, NU n = 394). B - Activation of adolescent abstinent CU compared to non-using controls, increased activation in CU shown in red (k = 5; CU n = 98, NU n = 106). C - Activation of adolescent abstinent CU compared to current adult and adolescent CU, increased activation in abstinent users shown in red (CCU: k = 22, n = 361; ACU: k = 5, n = 98). D - Adolescent current CU compared to non-using controls, increased activation in CU shown in red (k = 4; CU n = 69, NU n = 70).

All studies qualifying for the ACU group, were carried out in adolescent users. Further meta-analysis comparing ACU and CCU, using only adolescent studies was completed. Data from three manuscripts reporting on current adolescent users, with four separate comparisons, with 69 CCU and 70 NU were used for comparison with the abstinent adolescent user group described above. Results for all meta-analyses are reported in Table 3 and Fig. 2.

**Table 3:** Results from meta-analyses.


x	y	z	Voxels	p	SDM-Z	Egger’s test p value	Brain regions
Meta-analysis: CCU vs NU (k = 22; CU n = 361, NU n = 394)							
CCU > NU							
−4	−4	62	177	0.001409173	1.617	0.872	Left medial frontal gyrus extending bilaterally
38	18	2	340	0.000125647	1.942	0.406	Right insula extending to ipsilateral inferior frontal gyrus
CCU < NU							
−10	−98	−8	684	0.000094950	−1.664	0.302	Left cuneus extending to ipsilateral superior, middle, and inferior occipital gyri
30	−18	56	165	0.001311898	−1.288	0.595	Right precentral gyrus
					
Meta-analysis: ACU vs NU (only adolescent studies) (k = 5; CU n = 98, NU n = 106)							
ACU > NU							
46	−46	50	669	0.00013572	1.554	0.418	Right inferior parietal lobule extending to ipsilateral superior parietal and angular gyri
38	52	10	142	0.001023412	1.162	0.851	Right middle frontal gyrus extending to ipsilateral superior frontal gyrus
26	−92	6	86	0.00138104	1.121	0.652	Right middle occipital gyrus extending to ipsilateral superior occipital gyrus and cuneus
−34	58	−6	67	0.00138104	1.121	0.724	Left middle frontal gyrus extending to ipsilateral superior frontal gyrus
8	−60	68	56	0.000980318	1.170	0.816	Right precuneus extending to ipsilateral superior parietal gyrus
−42	−52	58	30	0.00138104	1.121	0.577	Left inferior parietal lobule extending to ipsilateral superior parietal gyrus
60	16	8	19	0.001511097	1.110	0.132	Right inferior frontal gyrus
Meta-analysis: CCU vs ACU (adult as well as adolescent studies) (CCU: k = 22, n = 361; ACU: k = 5, n = 98)							
ACU > CCU							
46	−48	50	390	0.000501096	1.244	0.443	Right inferior parietal lobule extending to ipsilateral inferior parietal, superior parietal and angular gyri
−10	−98	−8	263	0.000559688	1.228	0.320	Left lingual gyrus extending to ipsilateral middle and superior occipital gyri
6	−60	68	100	0.000584483	1.220	0.898	Precuneus extending to ipsilateral postcentral and superior parietal gyri
−34	58	−4	53	0.002155423	1.019	0.753	Left middle frontal gyrus extending to ipsilateral superior frontal gyrus
−38	−52	60	46	0.001968861	1.033	0.753	Left inferior parietal lobule extending to ipsilateral superior parietal gyrus
Meta-analysis: Adolescent CCU vs NU (k = 4; CU n = 69, NU n = 70)							
Adolescent CCU > NU							
44	28	34	220	0.000734389	1.061	0.238	Right middle frontal gyrus extending to ipsilateral inferior frontal gyrus.
40	−86	20	71	0.000344992	1.211	0.678	Right middle occipital gyrus

CCU: Current cannabis users; ACU: Abstinent cannabis users; NU: Non-user healthy controls.

### 3.2 Adult and adolescent CCU compared to NU

Meta-analysis of CCU revealed that CCU had increased activation when compared to NU in the medial frontal gyrus bilaterally and right insula, extending ipsilaterally to the inferior frontal gyrus. A decrease in activation was found in CCU compared to NU in the left cuneus, extending ipsilaterally to the superior, middle, and inferior occipital gyri; and in the right precentral gyrus, (k = 22; CU n = 361, NU n = 394).

### 3.3 Adolescent ACU compared to NU

Meta-analysis of adolescent ACU compared to NU revealed that ACU had increased activation in the right inferior frontal gyrus; right precuneus, extending ipsilaterally to the superior parietal gyrus; and the right middle occipital gyrus, extending ipsilaterally to the superior occipital gyrus and cuneus; the middle frontal gyrus extending to the superior frontal gyrus bilaterally and the inferior parietal gyri bilaterally, extending to the superior parietal gyrus bilaterally and the right angular gyrus. There were no areas where brain activation was significantly decreased in ACU compared to NU (k = 5; CU n = 98, NU n = 106).

### 3.4 Adult and adolescent CCU compared to adolescent ACU

Meta-analysis comparing CCU including both adult and adolescent studies to adolescent ACU, revealed no areas where brain activation was significantly increased in CCU compared to ACU, However, ACU had increased activation in the right precuneus, extending ipsilaterally to postcentral and superior parietal gyrI; the left lingual gyrus, extending ipsilaterally to superior and middle occipital gyri; the left middle frontal gyrus extending ipsilaterally to superior frontal gyrus; and the inferior parietal lobule bilaterally extending to superior parietal gyrus bilaterally and to the right angular gyrus, (CCU: k = 22; n = 361; ACU: k = 5; n = 98).

### 3.5 Adolescent CCU compared to NU

Meta-analysis of adolescent CCU revealed that adolescent CCU had increased activation when compared to NU in the right middle frontal gyrus extending ipsilaterally to the inferior frontal gyrus ; and the middle occipital gyrus on the right side. There were no areas where brain activation was significantly decreased in adolescent CCU compared to (k = 4; CU n = 69, NU n = 70).

### 3.6 Adolescent CCU compared to adolescent ACU

Meta-analysis of adolescent CCU compared to adolescent ACU, revealed that there were no areas where brain activation was significantly different between adolescent CCU and adolescent ACU NU (CCU: k = 4; n = 69; ACU: k = 5; n = 98).

### 3.7 Study heterogeneity

Funnel plots were created and examined for each cluster from each meta-analysis. Egger’s tests were used with no cluster reaching significance, indicating no publication bias (see Table 3). SDM-Z scores for each peak are reported in Table 3, no between-study heterogeneity was seen in the adolescent only ACU vs NU, CCU vs NU and ACU vs CCU meta-analyses. In the mixed age group comparison of ACU vs CCU, some heterogeneity was seen in the cluster spanning the left superior frontal gyrus.

## 4 Discussion

The key comparisons of interest in the present set of analyses relate to the brain activation differences between ACU and NU and that between ACU and CCU. Although the ACU versus NU comparison was limited to only studies in adolescent cannabis users (as no studies in adult users met our stringent inclusion criteria for ACU), we found significantly greater activation in the dorsolateral and ventrolateral prefrontal and posterior parietal cortices, which are part of the central executive network and are known to be involved in higher order cognitive processes such as attentional control, executive function and working memory (Seeley et al., 2007; Sridharan et al., 2008). These findings are similar to those found by a study in adolescent cannabis users following an abstinence period of average 5 weeks (Jager et al., 2010). Furthermore, ACU also displayed greater activation compared to NU in regions that are part of the default mode network (Buckner et al., 2008) such as the cuneus, inferior parietal cortex and angular gyrus as well as the visual cortex. Comparison of ACU and CCU across all studies (both adult and adolescent) revealed greater activation in ACU across regions within the central executive (dorsolateral prefrontal and posterior parietal cortices) and default mode (inferior parietal cortex and precuneus) network as well as lingual and precentral gyri. However, these differences were likely a result of CCU group including both adult and adolescent studies with ACU group including only adolescent studies. Direct comparison between ACU and CCU limited to studies in adolescent users alone revealed no significant group differences in brain activation. Further comparisons between CCU and NU across all eligible studies revealed activation differences in certain brain regions (such as inferior frontal gyrus where CCU > NU; and superior, middle and inferior occipital and precentral gyri where CCU < NU) that were broadly consistent with results from comparisons between all cannabis users and non-users in our previous meta-analysis (Blest-Hopley et al., 2018). Although, no comparably consistent patterns emerged from meta-analysis of adolescent studies alone, it is worth noting the limited number of studies eligible for inclusion for this comparison. Nevertheless, the robustness of our findings was supported by the results of the heterogeneity analysis, which showed no between-study heterogeneity in these clusters, as well as the results of the Egger’s test, which also showed that none of the reported clusters were significantly affected by publication bias.

Collectively, results of the present meta-analysis clearly suggest that at least in adolescent cannabis users, brain functional alterations persist even after periods of abstinence equivalent to around 25 days, by when cannabis metabolites are no longer detectable in urine. One cannot be certain on the basis of present analyses whether similar functional alterations also persist in adult regular cannabis users after comparable periods of abstinence. However, functional alterations in similar brain areas as reported here have also been observed in adult occasional cannabis users compared to non-users after prolonged abstinence (Colizzi et al., 2018a, b). Adolescence is a period of particular vulnerability to the effects of exogenous insults (Andersen, 2003; Belue et al., 1995; Rice and Barone, 2000; Spear, 2007) such as from use of drugs like cannabis, especially in light of progressive change in the density of cannabinoid receptors (Biegon and Kerman, 2001; Glass et al., 1997; Mato et al., 2003). Therefore, results presented here specifically underscore the particular vulnerability of the adolescent brain to residual effects of long-term cannabis use even after the drug and its metabolites have been fully excreted from the body.

What might underlie these functional differences between abstinent adolescent cannabis users and non-users? Evidence from two independent studies suggest downregulation of cannabinoid receptor 1 density in regular cannabis users (D’Souza et al., 2016; Hirvonen et al., 2012), which returns to normal levels following comparable periods of abstinence (D’Souza et al., 2016; Hirvonen et al., 2012), with normalisation starting as early as following 2 days of abstinence (D’Souza et al., 2016). This may suggest that functional alterations in abstinent adolescent cannabis users are unlikely to be related to altered availability of cannabinoid type 1 receptors. Whether these effects are related to longer-term alterations in glutamatergic (Colizzi et al., 2016) or dopaminergic (Sami et al., 2015) neurotransmitter systems also known to be affected by cannabis remains to be tested. The cross-sectional nature of the studies included in the present meta-analyses precludes inference regarding the precise nature of changes detected, whether they are a cause or consequence of cannabis use. Future, studies adopting longitudinal and genetically informed designs are necessary to disentangle causal factors from consequential effects (Paul and Bhattacharyya, 2018). Additional limitations need to be carefully considered while interpreting these results. Most importantly, this meta-analysis was limited by the number of studies available for inclusion. As a result, all studies meeting criteria for the ACU group contained only adolescent participants. As no adult studies met our criteria for inclusion to the abstinent group, it is therefore difficult to infer that functional alterations in adult users similarly persist following longer periods of abstinence. Results of comparisons between CCU and NU are also limited by inclusion of both adult and adolescent studies rather than separate analyses, in light of potentially different effects in these age groups (Blest-Hopley et al., 2018). However, this does not affect the key results of ACU vs NU and ACU vs CCU in adolescent only studies. Furthermore, the three papers investigating adolescent ACU were reported from the same research group, with almost complete overlap in the cohorts reported in two of the studies (Schweinsburg et al., 2008; Tapert et al., 2007) and around 45% overlap with a much larger cohort reported by Schweinsburg et al. (2011). Therefore, it may be argued that the meta-analysis results are inflated on account of non-trivial overlap of the samples investigated. However, it is also worth noting that these 3 studies reported on three distinct fMRI activation paradigms (Schweinsburg et al., 2008, 2011; Tapert et al., 2007). While this does not address the issue of overlapping samples, it suggests some degree of consistency across different cognitive tasks. Nevertheless, this highlights the limited nature of evidence currently available regarding longer term effects of cannabis that persist even after its metabolites are no longer detectable in the system, and underscore the need for further studies in this area. Another important limitation worth noting relates to the effects of withdrawal symptoms confounding comparisons including CCU. Again this is unlikely to have affected comparisons between ACU and NU as most withdrawal symptoms typically return to baseline by 2 weeks (Budney et al., 2004), while participants were abstinent for longer duration (25 days) in studies included in the ACU group. While some studies controlled for the potential confounding effects of other psychoactive substances (e.g. nicotine, alcohol) either statistically or by including study groups matched for use of these substances, others did not. Therefore, we cannot be completely certain that results of our meta-analyses were not influenced by potential effects of these substances on brain function, when they incorporated such studies that did not consider the confounding effect of comorbid exposure to other substances. Cannabis use parameters, such as age of onset and frequency of use, have been previously found to be important in relation to neurological changes (Broyd et al., 2016). A limited number of studies in the different sub-groups also precluded systematic examination of the relationship with the extent of previous cannabis use. The cannabinoid compositions of cannabis with regard to THC and cannabindiol levels has been found produce difference effects (Colizzi and Bhattacharyya, 2017), with high THC cannabis linked to increases in extreme psychological outcomes (Di Forti et al., 2009), no study has yet have reported cannabis type. Future studies may wish to further explore cannabis composition as a measure, alongside cannabis use parameters. Further to this, only certain types of cognitive processes, due to the limited number of studies, may have biased the results based on the cognitive processes used. The tasks employed by the adolescent ACU and CCU both included go/no go task, but the ACU group studies reported on two memory-related tasks as well, whereas the CCU had gambling and finger tapping tasks. While this may have introduced bias (Ganzer et al., 2016), it is worth noting that comparison groups (NU) were studied with the same tasks. Nevertheless, we cannot rule out this possibility. Methodological heterogeneity between studies, such as cannabis use levels, selective reporting of only results that reached statistical significance as is common practice, and studies with small sample sizes, are also caveats that are worth considering while interpreting these results.

Notwithstanding limitations highlighted above, results from the present meta-analyses suggest that certain neurofunctional alterations in components of the central executive and default mode networks in adolescent cannabis users may persist even after cannabis and its metabolites are likely to have left their bodies. However, given the overlap in the samples that support the key conclusions reported here, there is an urgent need for independent studies investigating whether brain function in abstinent cannabis users differ significantly from non-users following a sustained period of monitored abstinence, both in adolescents as well as in adults. Furthermore, whether these persistent brain functional alterations underlie short-term or longer-term risks of mental, social and behavioural disturbances (Boden et al., 2017; Fergusson et al., 2015; Sami and Bhattacharyya, 2018; Schoeler et al., 2018, 2016b; Silins et al., 2015, 2018), particularly in young people remains to be tested.

## Role of the funding source

The views expressed are those of the authors and not necessarily those of the NHS, the NIHR or the Department of Health. The funders had no role in the design and conduct of the study; collection, management, analysis, and interpretation of the data; preparation, review, or approval of the manuscript; and decision to submit the manuscript for publication. All authors have approved the final version of the paper.

## Financial disclosures

None.
